# Evaluation of dynamic infrared thermography as an alternative to CT angiography for perforator mapping in breast reconstruction: a clinical study

**DOI:** 10.1186/s12880-016-0144-x

**Published:** 2016-07-15

**Authors:** Sven Weum, James B. Mercer, Louis de Weerd

**Affiliations:** Medical Imaging Research Group, Department of Clinical Medicine, UiT The Arctic University of Norway, 9037 Tromsø, Norway; Department of Radiology, University Hospital of North Norway, Sykehusveien 38, P.O. Box 103, 9038 Tromsø, Norway; Department of Plastic Surgery and Hand Surgery, University Hospital of North Norway, P.O. Box 66, 9038 Tromsø, Norway

**Keywords:** Deep Inferior Epigastric Perforator (DIEP), Medical thermography, Perforator flap surgery, Perforator imaging, CT angiography, Radiology, Plastic surgery, Reconstructive surgery, Breast reconstruction

## Abstract

**Background:**

The current gold standard for preoperative perforator mapping in breast reconstruction with a DIEP flap is CT angiography (CTA). Dynamic infrared thermography (DIRT) is an imaging method that does not require ionizing radiation or contrast injection. We evaluated if DIRT could be an alternative to CTA in perforator mapping.

**Methods:**

Twenty-five patients scheduled for secondary breast reconstruction with a DIEP flap were included. Preoperatively, the lower abdomen was examined with hand-held Doppler, DIRT and CTA. Arterial Doppler sound locations were marked on the skin. DIRT examination involved rewarming of the abdominal skin after a mild cold challenge. The locations of hot spots on DIRT were compared with the arterial Doppler sound locations. The rate and pattern of rewarming of the hot spots were analyzed. Multiplanar CT reconstructions were used to see if hot spots were related to perforators on CTA. All flaps were based on the perforator selected with DIRT and the surgical outcome was analyzed.

**Results:**

First appearing hot spots were always associated with arterial Doppler sounds and clearly visible perforators on CTA. The hot spots on DIRT images were always slightly laterally located in relation to the exit points of the associated perforators through the rectus abdominis fascia on CTA. Some periumbilical perforators were not associated with hot spots and showed communication with the superficial inferior epigastric vein on CTA. The selected perforators adequately perfused all flaps.

**Conclusion:**

This study confirms that perforators selected with DIRT have arterial Doppler sound, are clearly visible on CTA and provide adequate perfusion for DIEP breast reconstruction.

**Trial registration:**

Retrospectively registered at ClinicalTrials.gov with identifier NCT02806518.

## Background

Breast reconstruction with a deep inferior epigastric perforator (DIEP) flap utilizing skin and subcutaneous tissue from the patient’s lower abdomen has become a popular option for women treated for breast cancer. The DIEP flap receives its blood supply from a perforator consisting of an artery and one or two comitant veins arising from the deep inferior epigastric artery (DIEA) and vein [1, 2]. In DIEP breast reconstruction the blood supply to the DIEP flap is reestablished by anastomosing the perforator to the internal mammary vessels.

The selected perforator is crucial for flap survival as it is the only source of blood supply to the flap. Although intraoperative perforator selection without preoperative perforator mapping is possible, the large variability in the numbers, locations and diameters of perforators makes this rather difficult [3–5]. A multicenter consensus study considered CTA the preferred method for preoperative perforator mapping [6]. CTA allows for precise anatomical description of the origin of perforators, their intramuscular course and point of fascia penetration. The main disadvantages of CTA are exposure to ionizing radiation and the use of intravenous contrast medium. The use of CTA is also associated with high costs. CTA can be time consuming due to delays in obtaining CTA preoperatively leading to delay in surgery, time the patient has to expend to obtain the CTA, and time of the surgeon and radiologist to review the imaging.

In 1993 Itoh and Arai described for the first time in English literature the use of dynamic infrared thermography (DIRT) for perforator mapping in DIEP flaps [7, 8]. Perforators that transport blood to the subdermal plexus cause a local heating at the skin surface that can be visualized as hot spots on infrared images. In DIRT a cold challenge is applied to the skin surface and temperature changes at the hot spots during the rewarming period are registered with an infrared camera. It might be beneficial for patients if DIRT could replace CTA in preoperative perforating mapping as DIRT, unlike CTA, does not involve the exposure to ionizing radiation or the use of intravenous contrast medium. DIRT has been used in the preoperative planning as well as intraoperative and postoperative monitoring of flap perfusion [9–14]. To our knowledge there are no studies that systematically have compared DIRT with other techniques for preoperative perforator mapping.

In this study the results of preoperative perforator mapping in DIEP breast reconstruction with DIRT were compared with those obtained with the most frequently used techniques hand-held Doppler and CTA. As flap survival is dependent on the selected perforator, all breast reconstructions were based on the perforator selected with DIRT and the surgical outcome was evaluated.

## Methods

This prospective clinical study was approved by the Regional Committee for Research Ethics. After giving informed consent to participation and publication of data, 25 women with mean age 57 years (range 38–69) and mean body mass index 27.2 kg/m^2^ (range 21.6–32.4) scheduled for DIEP breast reconstruction were included. Perforator mapping on the lower abdomen was performed with hand-held Doppler, DIRT and CTA in the same supine position. In all cases the DIEP breast reconstruction was based on the perforator selected with DIRT. The DIRT results were compared to the results obtained with the hand-held Doppler and CTA. Evaluation of the surgical outcome related to flap survival was made.

**Fig. 1:**
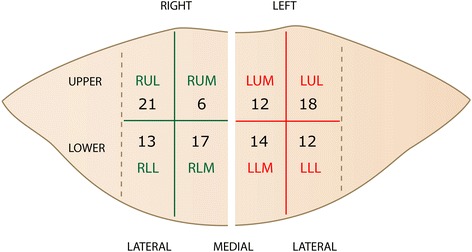
The quadrant system: The flap surface over each rectus fascia is divided into four quadrants. The abbreviations indicate right/left (R/L), upper/lower (U/L) and lateral/medial (L/M). The numbers indicate the distribution of hot spots in the 25 patients

The flap was marked on the lower abdomen. The lateral border of each rectus abdominis muscle was marked following palpation before and during muscle contraction. To describe the locations of perforators, a quadrant system was used. The flap surface overlying each rectus fascia was divided into 4 quadrants (Fig. 1). The vertical line at the midline between the upper and lower border of the flap is bisected in two equal lengths and defines the horizontal line between upper and lower quadrants. The area between the lateral border of each rectus abdominis muscle and the linea alba is bisected in equal parts by a vertical line.

Hand-held Doppler (8 MHz, Multi Dopplex II, Huntleigh Healthcare, Cardiff, UK) was used to locate arterial Doppler sounds within the quadrants and these were marked as black dots on the skin. DIRT included a 5-min acclimatization of the exposed abdomen at room temperature (22–24 °C).

**Fig. 2:**
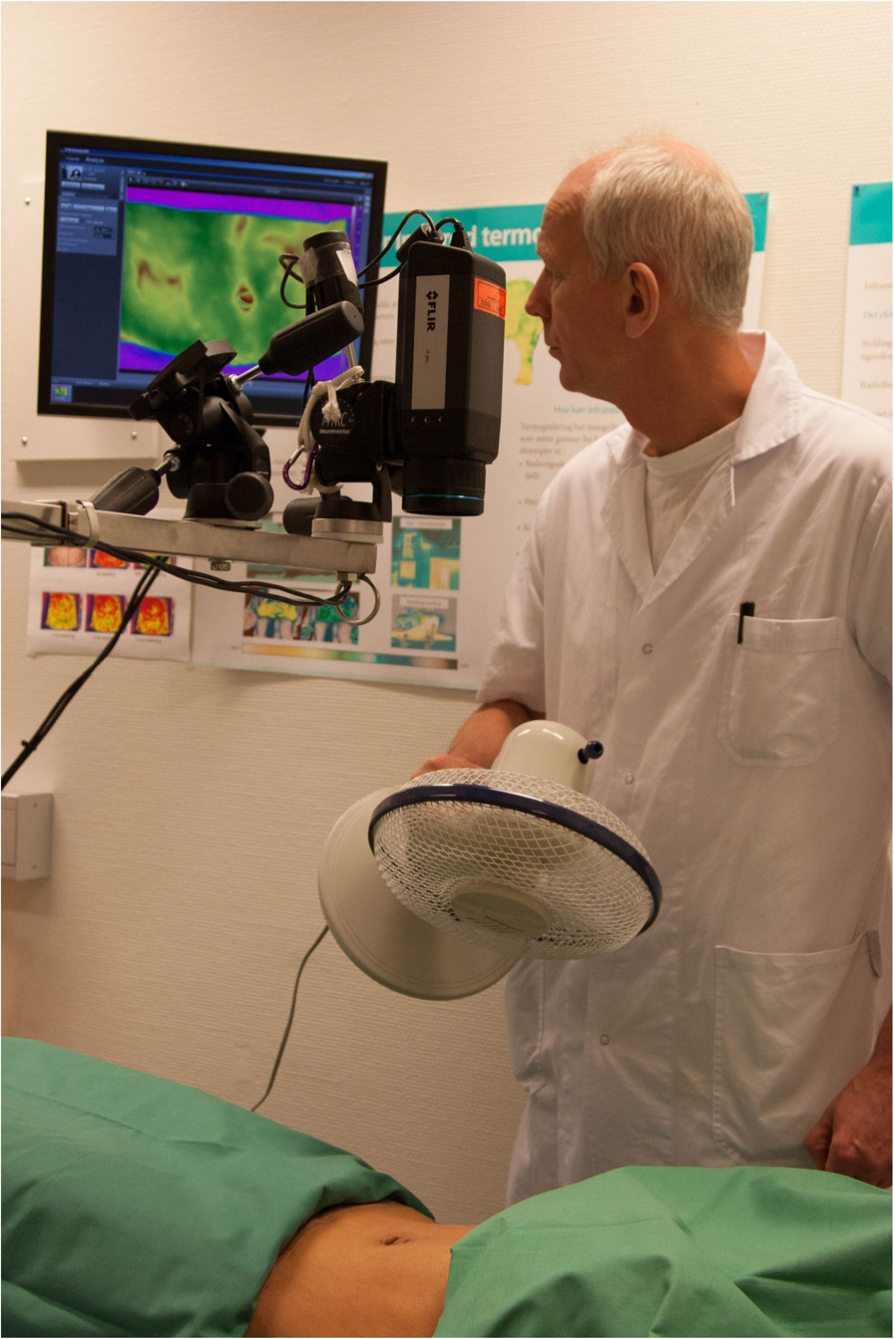
An infrared camera captures video sequences of thermal images before, during and after a cold challenge delivered by a desktop fan

**Fig. 3:**
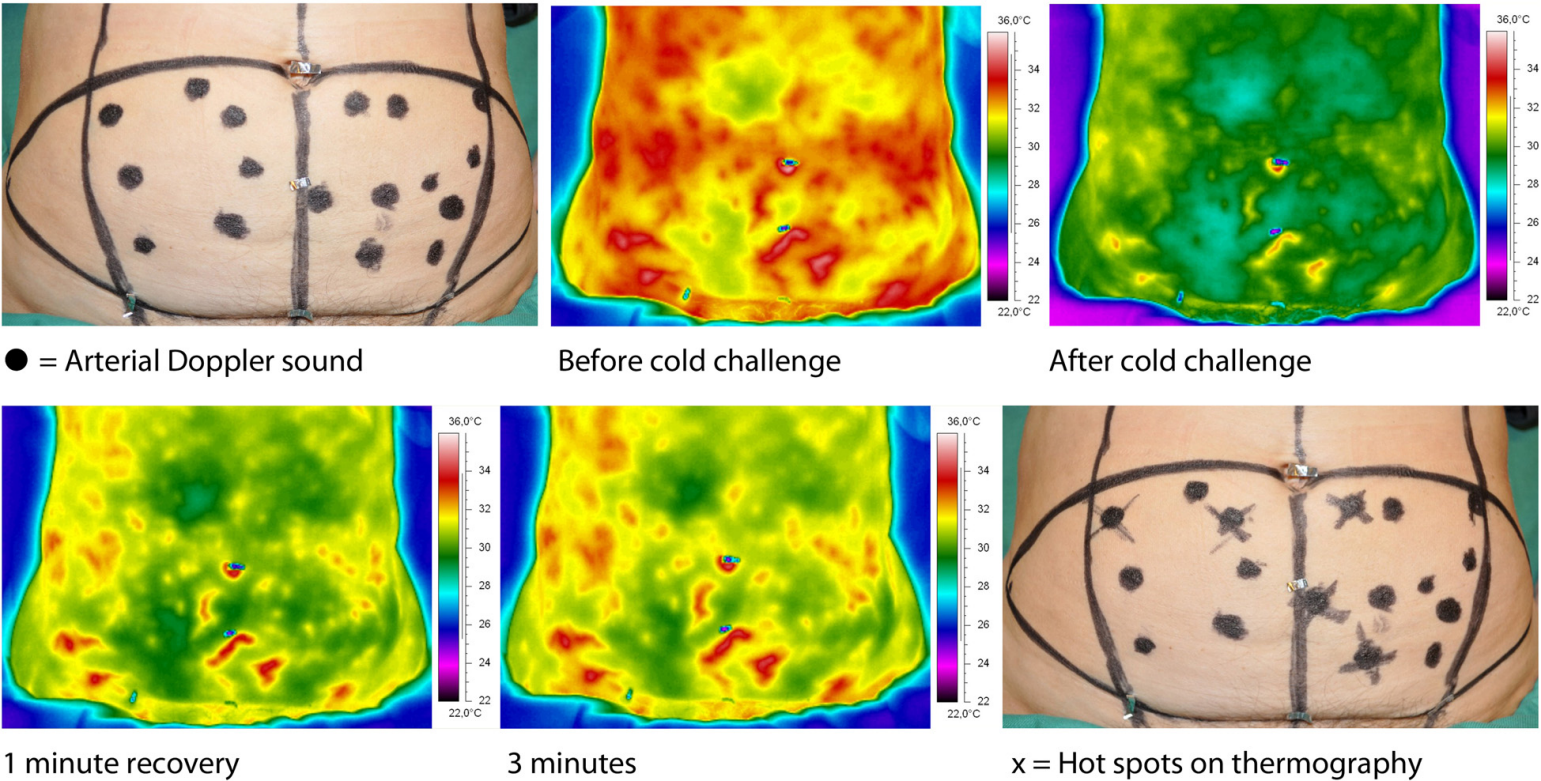
Black dots indicate locations of arterial Doppler sounds. After the cold challenge, some hot spots appear more rapidly and have a more profound rewarming. First appearing hot spots were associated with arterial Doppler sounds. The first appearing hot spots are marked with a cross and coincide with the location of an arterial Doppler sound. Some hot spots are located outside the quadrants and are not arising from the DIEA system. Only hot spots within the quadrants are marked

An infrared camera (FLIR ThermaCAM S65 HS, FLIR Systems, Boston, MA) was used to capture video sequences of thermal images before, during and after exposure of the lower abdomen to a cold challenge. This cold challenge was provided by blowing air at room temperature for 2 min over the abdomen using a desktop fan (Fig. 2). The temperature changes were well within the physiological range. After a recovery period of 3 min, the presence of arterial Doppler sounds at the first appearing hot spots was evaluated with hand-held Doppler. If present at a hot spot, its location was marked with a cross on the skin. A digital photo of the abdomen was taken at the end of the DIRT examination using the same angle as the infrared camera (Fig. 3). Thermal images and photos were stored on the hospital’s picture archiving and communication system (PACS).

The thermal images were qualitatively analyzed for the rate and pattern of rewarming of the hot spots. The first appearing hot spots and their associated quadrants were registered and named with increasing identification numbers based on their order of appearance. Hot spots showing the same rate of rewarming were ranked on basis of rewarming of the area around the hot spot. The hot spot with most progressive rewarming was given the lowest number.

CTA was performed (SOMATOM Sensation 16, Siemens Medical Solutions, Erlangen, Germany) after intravenous injection of contrast medium (Ultravist 370, Schering AG, Berlin, Germany or Iomeron 350, Bracco, Milan, Italy) with bolus triggering on the distal aorta. Scan parameters are summarized in Table 1. Three- dimensional (3D) and multiplanar reconstructions (MPR) were used to evaluate the DIEA and its perforators within the flap area (OsiriX version 4.0, OsiriX foundation, Geneva, Switzerland). On coronal thick maximum intensity projection (MIP) images the course and ramifications of the DIEA were evaluated on both sides. Axial thick MIP images were used to evaluate if there was a dominating DIEA system with perforators suitable for a DIEP reconstruction.

**Table 1:** CT scan parameters

Patient position: Head first supine	
Range: 3 cm cranial of umbilicus to symphysis	
Bolus tracking: Abdominal aorta 2 cm cranial to bifurcation	
Contrast medium: 120 mL Ultravist 370 or Iomeron 350, 4.0 mL/s	
Voltage: 120 kV	
Current: 150–200 mA	
Slice collimation: 0.75 mm	
Kernel: B20f medium	
Slice width/increment: 1.0 mm/0.7 mm reconstruction	

CTA images were analyzed by consensus between a radiologist (SW) and plastic surgeon (LdW). MPR images were used to see if the locations of the first appearing hot spots could be related to perforators from the DIEA. Axial images were used to decide if perforators were classified as lateral or medial. Sagittal images were used to decide if perforators were cranially or caudally located to the level midway between the umbilicus and the pubic symphysis.

## Results

### Preoperative results

**Fig. 4:**
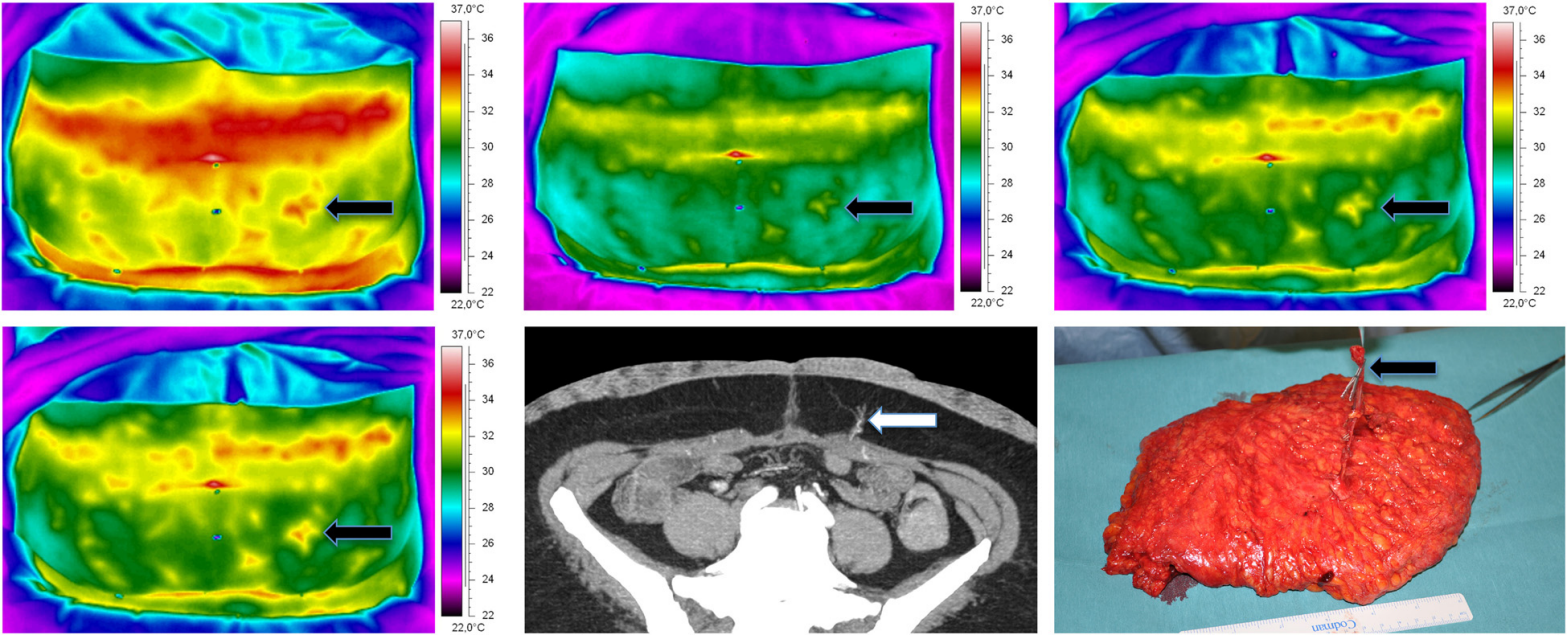
The selected hot spot (*arrow* on IR images) is associated with a perforator on CTA (arrow on CTA image) and intraoperatively (*arrow* on intraoperative flap image)

DIRT revealed a large variability in location, size, and number of hot spots between patients. This variability was also seen between the left and right side. Hot spots were always associated with arterial Doppler sounds. First appearing hot spots during rewarming were brighter than those appearing later. The hand-held Doppler does not allow for quantitative volume registration but, subjectively, the brightness of hot spots was related to the volume of Doppler sounds. All first appearing hot spots were also associated with clearly visible perforators on CTA and located in the same quadrants (Fig. 4). Hot spots were always slightly laterally located to the exit points of the perforators through the rectus fascia as seen on CTA.

In our 25 patients 113 hot spots were registered using DIRT and 108 (95.6 %) corresponded to a perforator in the same quadrant seen on CTA (Fig. 1). The remaining 5 hot spots (4.4 %) were all found in the lower part of the lower quadrants and corresponded to perforators coming from other arteries than the DIEA. The 113 hot spots were evenly distributed between the right and left side (57/56), as well as between the upper and lower quadrants (57/56). In the upper quadrants there were fewer medial than lateral hot spots (18/39).

Some large periumbilical perforators on CTA were associated with arterial Doppler sounds but not with hot spots. In these cases 3D reconstruction revealed a connection between the perforator and the superficial inferior epigastric vein in the periumbilical area. Not all arterial Doppler sounds locations could be associated with a hot spot on DIRT or a perforator on CTA.

### Surgical results

In all cases, the selected hot spots could be related to perforators found intraoperatively. Large periumbilical perforators that were not associated with a hot spot consisted of a small artery with one or two large comitant veins. The marked hot spots were always slightly laterally located to the exit points of the perforator through the fascia. While all first appearing hot spots could be related to suitable perforators, not all arterial Doppler sound locations on the skin could be related to suitable perforators intraoperatively. All flaps were based on the selected perforator from DIRT and were adequately perfused. Of the 25 flaps, 24 survived. One flap was lost on the second postoperative day due to a bleeding beneath the flap that was diagnosed too late to save the flap. This complication could not be related to the selected perforator. The mean flap weight was 713 grams (range 302–1270). Twelve flaps were based on one perforator, nine flaps on two perforators, two flaps on three perforators and two flaps on four perforators. In all cases the selected perforator visualized with DIRT was the most suitable perforator, additional perforators were added in cases with large volume flaps to guarantee adequate flap perfusion.

## Discussion

The main finding of this study is that the first appearing hot spots on DIRT were always associated with arterial Doppler sounds as well as clearly visible perforators on CTA and intraoperatively. The use of DIRT in preoperative perforator mapping provided a suitable perforator for DIEP breast reconstruction in all 25 cases.

Surgeons want information on the hemodynamic properties of a perforator as well as its location. Hand-held Doppler is frequently used for preoperative perforator mapping. Giunta et al. attributed the high number of false-positive results in their study to the relative high sensitivity of the hand-held Doppler [15]. Very small perforating vessels were also located, unsuitable for perforator flaps because of their narrow caliber. Others have abandoned hand-held Doppler because the results often proved to be aberrant from the intraoperative observations [16]. Similar to Giunta we found arterial Doppler sounds that could not be associated with hot spots, nor with suitable perforators intraoperatively.

CTA has become the current gold standard for perforator mapping and is based on the perfusion of the perforators with contrast medium during the arterial phase. It provides information on the caliber of each perforator, its intramuscular course and its exit point through the anterior rectus fascia [6]. However, a recent study by Cina et al. revealed that the sum of the diameter of the perforating artery and vein with color Doppler was in agreement with the diameter of the presumed artery on CTA [2]. However, there was a significant disagreement between the measured diameters of the arteries measured with color Doppler and CTA, as well as for CTA and intraoperative findings. Thus, measurement of the assumed perforating artery on CTA may in fact constitute the sum of the diameters of the perforating artery and vein(s). Mathes et al. warned against sole reliance on CTA perforator mapping as they had to make a significant number of changes intraoperatively [17]. Important disadvantages of CTA are exposure to ionizing radiation and the use of intravenous contrast medium. A method without these disadvantages would be beneficial.

In our study, the rate and pattern of rewarming of the hot spots were analyzed. There was a large variability in the number of hot spots and in the rate and pattern of rewarming. As first appearing hot spots were associated with perforators on CTA and arterial Doppler sounds, the rewarming at the hot spot is a result of blood perfusion through the perforator to the skin surface.

During the rewarming period all perforators compete with each other in skin rewarming. A rapid rewarming indicates that the associated perforator transports more blood to the skin surface than a perforator that produces a slower rewarming. Progressive rewarming around the hot spot indicates a well-developed vascular network around this hot spot. A well-developed branching pattern is also considered an important criterion when selecting a perforator on CTA [18, 19]. By analyzing the rate and pattern of rewarming, the surgeon obtains information on the perforator’s hemodynamic properties.

Information on the location where the suitable perforator can be found is of great value to the surgeon. Chubb et al. reported their preliminary results with DIRT and reported that this technique matched the accuracy of CTA for perforator location [20]. We found, however, that the hot spot on the skin was always slightly laterally located in relation to the perforator’s exit point through the rectus abdominis fascia. Using CTA, Rozen et al. found in their perforator angiosome study that lateral row perforators have a long and laterally directed course, whereas the medial perforators show a straighter course from the rectus sheath towards the skin [21]. An explanation for this lateral orientation of vessels was given by the eminent anatomist John Hunter (1728–1793), who saw it as a product of growth that occurs from the stage of fetus to adulthood [22]. In contrast to CTA, DIRT does not provide information on the intramuscular course of the perforator. Although the main goal of perforator mapping is to find a perforator that can provide adequate blood perfusion of the flap, a perforator with a short intramuscular course is preferred to a perforator with the same caliber and a longer intramuscular course. Interestingly, de Weerd et al. found in a DIRT study that first appearing hot spots were often associated with perforators passing through the tendineous intersection [19]. These perforators have a very short intramuscular course. A further advantage of CTA over DIRT is that CTA can provide information on the continuity of the deep inferior epigastric system in patients that have been previously operated in that area. In cases with large flap volumes, additional perforators were added to the selected perforator to optimize flap perfusion, as also reported by Gill et al. in their retrospective study [23].

Our results showed cases in which large periumbilical perforators on CTA could not be associated with hot spots. This indicates that these perforators do not transport much blood the skin surface. We postulate that such perforators consist of a small caliber artery and one or two large caliber comitant veins and that these large veins communicate with the superficial inferior epigastric vein. This postulation is supported by our intraoperative findings and CTA. The existence of these communications was already described by Carramenha e Costa et al. in an anatomic study and nicely illustrated with the use of CTA by Rozen at al. in a study on the venous anatomy of the abdominal wall [24, 25].

One of the disadvantages of DIRT is that only perforators that transport blood to the skin surface are detected. It is possible that a perforator that ends in the subcutaneous tissue might be a suitable perforator for DIEP breast reconstruction. Such a perforator can be detected with CTA but not with DIRT. From earlier studies it is known that the subdermal plexus contributes to the perfusion of the underlying subcutaneous tissue. In their cross sectional radiographic study, Taylor et al. revealed blood supply to the subcutaneous layer caused by “raining down” from the subdermal plexus, a result confirmed by Schaverien et al. [26, 27]. Initially, the perfusion of the DIEP flap depends on this mechanism. During the first postoperative week, the interconnections between the vascular structures within the flap increase in size and, as a result, tissue perfusion improves [28]. The main limitation of this study is the small number of 25 patients. Because of the small study size our results are indicative and should be interpreted within the context of this limitation. One limitation in this study is that we were unable to objectively measure perfusion of subcutaneous tissue. Such is possible using indocyanine green angiography intraoperatively [29]. Inadequate perfusion of subcutaneous tissue fat may cause fat necrosis or wound-healing problems. None of our patients had a wound-healing problem at the reconstructed site or required a reoperation for fat necrosis. Another limitation is that DIRT was used in relatively healthy patients. The mean BMI was 27.2 kg/m^2^ (range 21.6–32.4). Further studies are required to evaluate the usefulness of DIRT in preoperative perforator mapping in patients with co-morbidities and obesity.

## Conclusions

We conclude that the locations of first appearing hot spots on DIRT are associated with arterial Doppler sounds and with perforators on CTA. In addition, the surgical results revealed that DIEP breast reconstruction could reliably be performed using DIRT for perforator selection. DIRT provides information on the location of the perforator and its hemodynamic properties. DIRT is easy to interpret and it does not involve exposure to ionizing radiation or the use of intravenous contrast medium. Based on our results we conclude that DIRT is a promising alternative to CTA for preoperative perforator mapping in DIEP breast reconstruction.

## Abbreviations

3D, Three-dimensional; CT, Computed tomography; CTA, Computed tomographic angiography; DIEA, Deep inferior epigastric artery; DIEP, Deep inferior epigastric artery perforator; DIRT, Dynamic infrared thermography; MIP, Maximum intensity projection; MPR, Multiplanar reconstruction; PACS, Picture archiving and communication system
